# Exploring the predictors of physical inactivity in a university setting

**DOI:** 10.1186/s12889-022-14953-5

**Published:** 2023-01-09

**Authors:** Lawrence Bismarck Ndupu, Mark Faghy, Vicki Staples, Sigrid Lipka, Chris Bussell

**Affiliations:** 1 School of Life Sciences, Faculty of Health and Life Sciences, Coventry University, 20 Whitefrairs Street, Coventry, CV1 2DS UK; 2 Human Sciences Research Centre, University of Derby, Kedleston Road, Derby, Derbyshire DE22 1GB UK

**Keywords:** Physical inactivity, Theoretical domains framework, University, Students, Staff, Predictors

## Abstract

**Background:**

Changes in lifestyle patterns and the dependence on technology have contributed to an increase in prevalence of inactivity. To address this there is a need to identify the predictors of physical inactivity using the Theoretical Domains Framework (TDF).

**Methods:**

One hundred and twenty-one university administrative staff and 114 PhD students completed a survey. Physical activity (PA) levels were assessed using the Global Physical Activity Questionnaire (GPAQ), with participants scoring below 600 MET-minutes/week of total PA regarded as inactive. The predictors of physical inactivity were assessed using the Determinants of Physical Activity Questionnaire (DPAQ). Multiple regression analyses were used to identify which domains of the TDF predicted physical inactivity in the study samples.

**Results:**

The results indicated that 64% of administrative staff (Mean = 411.3 ± 118.3 MET-minutes/week of total PA) and 62% of PhD students (Mean = 405.8 ± 111.0 MET-minutes/week of total PA) did not achieve the recommended PA levels. The physical skills domain (t _106_ = 2.198, *p* = 0.030) was the significant predictor of physical inactivity amongst the administrative staff. Knowledge (t _99_ = 2.018, *p* = .046) and intentions (t _99_ = 4.240), *p* = 0.001) domains were the significant predictors of physical inactivity amongst PhD students.

**Conclusions:**

The results of this study should be used as a theoretical starting point in carrying out behavioural diagnosis, which could inform the design of effective interventions to increase PA levels in universities and other settings.

## Background

Physical activity (PA) is acknowledged as a key determinant in the prevention and management of chronic diseases such as diabetes, hypertension, stroke, heart disease, and some cancers [1]. Regular PA also helps to maintain body weight and can enhance mental health and wellbeing, including important areas of quality of life [1, 2]. Despite the well documented benefits of PA, academic literature highlights that over a quarter (28%) of the world’s population are physically inactive [3], and do not meet the established PA guidelines of engaging in 150 minutes of moderate PA or 75 minutes of vigorous PA or a blend of both on a weekly basis, as well as engaging in muscle strengthening activities at least 2 days weekly [4]. Physical inactivity is a global concern and has been acknowledged as the fourth prominent cause of morbidity and mortality [3, 5], with reports of up to 5 million deaths annually.

Sedentarism (i.e. engaging is insufficient amount of PA) is becoming more prevalent in occupational settings due to advancements in technology and a shift towards less physically demanding jobs [6]. Occupational settings such as universities are unique settings to promote PA engagement because of the different roles for staff. Specific job roles may be subject to different guidelines as regards work-associated movement, such as being wholly desk-based [7]. The university setting also provides group support, existing structures of formal and informal interactions between staff and students, accessibility to staff and students and prospective shared behavioural standards, and easy access to onsite facilities and existing frameworks that can easily be enhanced to include staff, which are not typical in other places of employment. Therefore, all these factors are specific benefits of university-based interventions over those in other settings [8, 9]. In universities, an ideal opportunity is presented to easily access and encourage a considerable percentage (40%) of young students who are leaving their homes for the first time to adopt healthy lifestyles [10]. For example, in the UK alone, over 570,475 students enrolled to start an undergraduate course in 2020 [11]. Therefore, since the possibility of meeting the recommended PA guidelines progressively declines between 18 and 25 years [12] when several adolescents are in universities, interventions can be put in place to encourage the adoption of healthy lifestyle behaviours. This is important because previous studies suggest that the behaviours adopted by adolescents can transcend to adulthood [13].

Occupations are becoming more sedentary, with adults spending approximately 77% of their waking period physically inactive at work [14]. Therefore, in promoting PA, it is imperative to consider the amount of time people spend at work, because this has the likelihood to adversely impact on their opportunities for PA engagement and promote a fairly inactive lifestyle [15]. Likewise, in universities, staff and students generally spend nearly 60% of their waking time physically inactive [16]. This is reinforced by a study carried out in the UK which indicated that university students spent 8 hours daily engaging in inactive behaviours such as studying, playing games, using computers, hanging out with friends, watching TV, shopping, chatting and deskbound [17]. Therefore, any intervention aimed at improving PA levels among university staff and students would be beneficial in promoting more active lifestyles. Furthermore, universities offer opportunities to be active through the provision of bicycle sheds and changing facilities, as well as the provision of flexible working environments to give staff and students a sufficient degree of independence in managing their own time. Despite the opportunities inherent in universities to be active, the levels of physical inactivity among university staff and students remains high and thus a major concern [18].

Previous studies conducted in several countries reported high rates of physical inactivity amongst university staff and students. For example, Pengpid et al. [18] in a survey involving undergraduate students across universities in 23 low-, medium-, and high-income countries reported that 44.4% were physically inactive, which, they suggested, could be due to the lack of social support, low self-efficacy, low sense of control, overweight or obesity, lack of awareness about the association between PA and heart disease, and lack of conviction about the health benefits of PA [18]. Although university staff are a comparatively under-studied population compared to university students, Cooper and Barton [19] in a survey on PA and wellbeing of university staff in the UK suggested that 42.0% of all staff were physically inactive. Likewise, Rissel et al. [20] in a survey carried out among university staff in an Australian university reported that 69.0% were physically inactive. These findings are congruent with that from Faghy et al. [7], who reported that university staff experienced high levels of inactivity during both their working day, as well as their leisure time. These studies have reported differences in physical inactivity levels across universities in different countries, which has been attributed to the lack of standardisation of instruments used in assessing physical inactivity across studies, lack of consideration of within-group differences of roles and responsibilities, lack of theoretical basis to understand lasting change, and the focus on diverse populations in different settings [21]. Therefore, in using these data to offer any conclusions regarding physical inactivity levels among university staff and students, it is important to consider the diversity of populations, onsite facilities and cultures in the studies, which may have some effects on the results. However, even with these differences in results reported, it is evident that physical inactivity levels are high among university staff and students [18–20], and thus a major public health concern requiring theory-informed research to understand the predictors of physical inactivity in these populations, in order to inform interventions aimed at changing behaviours towards PA.

Identifying the predictors of physical inactivity and understanding the theoretical underpinning of behaviour change will better inform the development of effective strategies to change behaviour towards PA [22, 23]. However, the lack of theoretical underpinning in the approaches to understand and change behaviour has made it challenging for people to change their behaviour [24]. This is because changing behaviour is complex and involves personal, relational, environmental, cognitive and policy factors, which must be considered in designing effective behaviour change interventions [23], thus establishing the importance of supporting PA interventions with psychological theories. More focus should be placed on utilising theory to identify those factors that prevent university staff and students from engaging in PA in order to design effective interventions. Therefore, investigations that enhance the understanding of any of these factors have an increased likelihood to increase engagement with PA [24].

Health psychology provides various behaviour change theories that can be used in the university setting to help staff and students make changes to their daily lifestyle. Michie et al. [23, 25] developed a framework through the synthesis of 33 frequently employed theories of behaviour change and 128 psychological constructs, which was grouped into 14 domains [26], and is known as the Theoretical Domains Framework (TDF). The TDF has been recognised as a valuable instrument for identifying the factors that contribute to behaviour and barriers to behaviour change, because it presents a theoretical lens through which influences on behaviour (e.g. social, affective, mental, and environmental factors) can be examined [27]. This means that the TDF helps in identifying the determining factors of behaviour change which prospective interventions can focus on. Recently, there have been growing demands for more explicit utilisation of psychological frameworks such as the TDF to ascertain the influences on behaviour change, understand the mechanism through which change occurs, and inform implementation interventions [28–30]. However, to date, several studies have utilised the TDF [23] to investigate barriers to and enablers of PA and exercise among diverse populations in different settings [31–33], but to our knowledge, no study has applied the TDF to identify the predictors of physical inactivity in staff and/or student populations in a university setting. In addition, based on an unpublished survey data [34] suggesting that administrative staff and PhD students were the most physically inactive groups compared to other university staff and students groups, respectively, the current study focussed on these two groups.

Therefore, the aim of this study was to gain knowledge about the predictors of physical inactivity among inactive university administrative staff and PhD students, utilising the TDF to guide the exploration. It was hypothesised that the predictors of physical inactivity will differ between administrative staff and PhD students.

## Methods

### Study design

A prospective cross-sectional survey was conducted in a university in the United Kingdom. This study involved an open online survey administered to administrative staff and PhD students between February and April 2019.

### Ethical considerations

This study was approved by the Human Sciences Research Ethics Committee (HS-REC) of the University of Derby, Derby, United Kingdom (Ref no: 97–1718-LNs on 19/03/2018). The online survey included a participant information page, which detailed the purpose of the study, how participants’ anonymity and confidentiality would be ensured, and how the data generated would be securely stored and used. Participants were then required to consent to participating in the study before being allowed to complete the survey. All methods were carried out in accordance with relevant guidelines and with the Helsinki Declaration of 1975, as revised in 2000.

### Sampling

A power analysis carried out a priori with G* Power computer programme (version 3.1.9.2, Universität Düsseldorf, Germany), suggested that to use a multiple linear regression with alpha set at 0.05, at least 135 PhD students and administrative staff each (i.e., 270 participants) were needed to detect a moderate effect size (f2 = 0.15), with power of 80%. Multiple linear regression was used to examine the relationship between several predictor variables (14 domains of the TDF) and an outcome variable (physical inactivity).

Convenience sampling technique was utilised to recruit participants to take part in this survey study. All current administrative staff (i.e., non-academic staff who provide support, such as admissions, clerical work, maintenance of official records, safety and security, IT services, research administration, student services and public affairs), and students enrolled on a traditional PhD, Professional Doctorate, or PhD by publication who were 18 years and above were invited to take part in the study, as part of the screening process, to measure their PA levels.

An earlier survey (not reported in this paper) conducted among all university staff and students indicated that administrative staff (1330.3 ± 1253.89 MET-minute/week of mean physical activity) and PhD students (1305.9 ± 1001.09 MET-minute/week of mean physical activity) were the most physically inactive, in comparison with other staff and student groups, respectively. This study therefore focused on university administrative staff and PhD students, who are more likely to benefit from prospective interventions.

### Study procedure

The online survey was administered through Qualtrics (Q Plus, USA), a secure online platform. Participants gave informed voluntary consent to take part in the study and all data were anonymised. The survey took about 25 minutes to complete. Incentives were used to encourage survey completion by providing the opportunity for interested participants to enter a prize draw at the end of the survey, with the chance of winning a £50, £30, or £20 Amazon voucher. One each of these three categories of Amazon vouchers were available to both administrative staff and PhD students to be won, i.e., a total of £200 Amazon vouchers were given to the participants as incentives. Ethical approval was received from the Human Sciences Research Ethics Committee (HS-REC) of the University of Derby (Ref no: 09–1718-LNs on 19/03/2018).

### Materials

The survey was designed to assess university administrative staff and PhD students’ demographic characteristics, physical inactivity levels and perceived influences on PA engagement, to understand why they are physically inactive. Several validated questionnaires were used in this survey. The survey for the administrative staff and PhD students consisted of 71 items and 75 items, respectively. The survey was piloted among a sample of university administrative staff (*n* = 20) and PhD students (n = 20); based on the feedback received about clarity of instructions and items, no amendments were required.

### Outcome measures

#### Demographics

The sociodemographic information collected for the administrative staff and PhD students included gender, age, ethnicity, and employment status, as well as the study mode (i.e., traditional PhD, Professional Doctorate, or PhD by publication) for the PhD students.

#### Physical activity behaviours

The Global Physical Activity Questionnaire (GPAQ) [35] is a validated 16-item scale that measures moderate- and vigorous-intensity PA in three separate domains, i.e. work, leisure activities and active transportation domains, as well as sedentary behaviours. The GPAQ has been established as a valid and reliable instrument to measure PA among university staff and students [36–38]. Previous studies [36, 38] have established the excellent psychometric properties of the GPAQ, with its Kappa (0.67 to 0.73) and Spearman’s rho (0.67 to 0.81), varying from moderate to significant strength, respectively. In addition, the GPAQ has also been authenticated with objective tools used in measuring PA (e.g., accelerometers and pedometers) [39], and the most extensively utilised subjective measures for PA such as the International Physical Activity Questionnaire (IPAQ) [40]. Using the GPAQ scoring protocol, participants were grouped as physically active (i.e. scoring above 600 MET-minutes/ week of total PA) or physically inactive (i.e. scoring 600 MET-minutes/ week of total PA or below) [35].

#### Predictors of physical activity

The Determinants of Physical Activity Questionnaire (DPAQ) [41], a validated 34-item scale developed to measure the domains of the TDF in the PA context was used to measure the predictors of physical inactivity. Even though the DPAQ measures the TDF domains in the PA context, negative correlations between the domains of the TDF and PA can be used to predict physical inactivity. For example, lower physical skills score was associated with lower total self-reported PA (i.e., higher total physical inactivity) among the administrative staff. The DPAQ, however, measures only 11 of the 14 domains of the TDF. The ‘reinforcement’, ‘social/professional role and identity’ and ‘memory, attention & decision processes’ domains of the TDF are not measured by the DPAQ. Therefore, to test if the 14 domains of the TDF predict physical inactivity among university administrative staff and PhD students, other validated scales were used to measure these three domains. The psychometric properties of the DPAQ have been validated among university staff and students across the UK [41], with its Cronbach’s alpha coefficient (i.e. internal consistency) and test-retest reliability varying from 0.57 to 0.86 and from 0.45 to 0.91, respectively [41], reinforcing the selection of this scale in the present study. The items in this scale were evaluated with a 7-point Likert scale varying from 1- ‘Strongly agree’ to 7 = ‘Strongly disagree’. The scores of items in each subscale were then summed up and divided by the total number of items to get the mean score. This is a relatively new scale, and thus more validity tests are required.

#### Data analysis

Descriptive statistics were presented as means with standard deviations (SD) and percentages of the socio-demographic characteristics of the study participants, except otherwise indicated. Physical inactivity levels were computed using the WHO’s GPAQ protocol and reported as MET-minutes/week of total PA [35]. Based on the total MET-minutes/week of PA scores, participants were categorised as either physically active (i.e., MET-minutes/week of total PA ≥ 600) or inactive (i.e., MET-minutes/week of total PA < 600. The data from the physically inactive participants were then analysed to establish predictors of physical inactivity. Multiple regression analysis was used to assess the relationship between the 14 domains of the TDF (independent variables as measured by DPAQ, the intrinsic motivation subscale of the MPAQ and the six additional items) as predictor variables and total self-reported physical inactivity levels (as dependent variable) [42]. All data analyses were carried out using IBM Statistical Package of Science for Social Sciences (SPSS) Statistical Software, version 25.0 (SPSS Inc., Chicago, IL, USA), with significance levels set at 0.05.

### Results

#### Response rate

In total, 411 survey responses (i.e., 198 responses from PhD students and 213 responses from administrative staff who accessed the survey online) were collected, representing 78 and 35% of the PhD student and administrative staff populations, respectively. Of this, 184 PhD students and 189 administrative staff (i.e., 373 participants) fully completed the survey. Since the focus of this study was on physical inactivity, 138 respondents (i.e., 68 administrative staff and 70 PhD students) that scored above 600 MET-minutes/week on the GPAQ were regarded as physically active and removed from subsequent analysis. Therefore, following the removal of all participants that were physically active, 235 survey responses (i.e., 114 and 121 responses from university PhD students and administrative staff, respectively who scored below 600 MET-minutes/week) were included in the final analysis. The actual samples were close to the target numbers required for this study.

#### Demographics

The demographic characteristics of the participants are presented in Table 1. More than half of the PhD students (55%) were male, while more than three-quarters of the administrative staff (84%) were females. This is consistent with the gender split across faculties in the university where this study was conducted, with 57% of the PhD student population made up of males and 83% the administrative staff population made up of females.

**Table 1:** Socio-demographic features of university administrative staff and PhD students

Variables	Administrative staff N (%)	PhD students N (%)
Gender		
Male	19 (15.7)	63 (55.3)
Female	102 (84.3)	51 (44.7)
Age		
Young adults (18–35 years)	9 (7.4)	31 (27.2)
Intermediate adults (36–55 years)	99 (81.9)	79 (69.3)
Older adults (56 years and above)	13 (10.7)	4 (3.5)
Mean age	45.9 ± 7.01 years	39.9 ± 8.04 years
Ethnicity		
White	115 (94.3)	81 (71.1)
Black/African/Caribbean/Black British	4 (3.3)	14 (12.3)
Asian/Asian British	–	16 (14.0)
Mixed/Multiple ethnic groups	2 (1.6)	2 (1.8)
Other ethnic groups	1 (0.8)	–
Employment Status		
Full-time	101 (83.5)	–
Part-time	21 (16.5)	–
Study Mode		
Full-time	–	101 (83.5)
Part-time	–	21 (16.5)

#### Physical inactivity levels

The operational definitions for the 14 domains of the TDF are illustrated in Table 2. In order to identify the predictors of physical inactivity in university administrative staff and PhD students, multiple regression analyses were carried out to examine the contribution of the 14 domains of the TDF to physical inactivity levels. The total PA score as measured by the GPAQ was utilised as the dependent variable (i.e. outcome variable), while the independent variables (i.e. predictive variables) were: the total score of the DPAQ (measuring knowledge; physical skills; intentions; behavioural regulation; environmental, context and resources; beliefs about capabilities; beliefs about consequences; optimism; goals; social influences; and emotion domains of the TDF); total score of the MPAQ (measuring the reinforcement domain of the TDF); and total scores of the additional six items developed (measuring memory, attention, and decision processes; social/professional role and identity domains of the TDF). The normality probability plot indicated that the dependent variable was distributed normally. Other assumptions for multiple linear regression such as linearity (scatter plots for administrative staff and PhD students showed linear relationships between the predictor variables and outcome variable); multicollinearity (Variance Inflation Factor (VIF) for administrative staff ranged from 1.725–4.045) and PhD students ranged from 1.311–4.533); independence (Dublin-Watson value was 2.143 for administrative staff and 1.383 for PhD students); and homoscedasticity (plots of standardised residuals versus predicted values for administrative and PhD students exhibited a pattern) were all met.

**Table 2:** Operational definitions of the TDF domains

TDF Domains	Definition
Knowledge	Awareness about the benefits of PA, detrimental impacts of physical inactivity and the recommended PA guidelines
Physical Skills	Abilities or proficiencies gained via practice
Intentions	Deliberate resolve to carry out a behaviour or a determination to perform in a specific manner
Social Influences	Relational processes that can influence people to transform their views, state of mind, or conducts
Environmental Context and Resources	Circumstances of an individual’s condition or surrounding that hinders or promotes the enhancement of abilities and proficiencies, autonomy, social expertise, and adaptive demeanour
Beliefs about Consequences	Recognition of the fact, certainty, or authenticity concerning the after-effects of a behaviour in a specified circumstance
Beliefs about Capabilities	Recognition of the fact, certainty, or authenticity concerning a skill, facility, or proficiency that an individual can utilise constructively
Reinforcement	Heightening the possibility of a reaction by arranging a contingent association or unforeseen event, between the reaction and a specified incentive
Social/Professional Role and Identity	Rational series of behaviours and exhibited individual attributes of a person in a public or work environment
Memory, Attention and Decision Processes	Capability to keep information, selectively focus on parts of the environment and select amongst two or more options
Optimism	Confidence that things would turn out for the best or that anticipated targets would be achieved
Goals	Cognitive depiction of aftermath or end results that a person intends to attain
Emotion	Intricate response pattern, including empirical, physiological, and behavioural components, through which a person tries to handle an individually important issue or incident
Behavioural Regulation	Anything focused on controlling or transforming impartially perceived or assessed activities

NB: TDF- Theoretical Domains Framework [32].

In the multivariate model, as illustrated in Table 3, physical skills domain of the TDF was found to be the only significant predictor of physical inactivity among the university administrative staff, with the adjusted R^2^ = 0.25. Lower physical skills score (β = 31.22; 95% CI = 3.05–59.38; *p* = 0.03) was associated with higher total self-reported physical inactivity among the administrative staff.

**Table 3:** Multiple regression analysis for predictors of Physical Inactivity among administrative staff

Variables	β (95%CI of β)	*P* Value
Knowledge	2.43 (− 13.67–18.13)	0.759
Intentions	−27.23 (− 64.53–10.07)	0.151
Physical Skills	31.22 (3.05–59.38)	0.030
Goals	12.28 (−5.58–30.03)	0.176
Environmental Context and Resources	−2.35 (− 37.03–32.33)	0.893
Social Influences	7.27 (− 25.59-40.13)	0.662
Emotion and Reinforcement	4.13 (−32.98–41.23)	0.826
Beliefs about Capabilities	23.71 (−10.22–57.64)	0.169
Beliefs about Consequences	−37.96 (− 112.71–36.79)	0.316
Behavioural Regulation	11.63 (−0.52–23.78)	0.061
Cognitive and Interpersonal Skills	15.14 (−46.19–76.47)	0.626
Optimism	1.04 (−26.51–28.59)	0.941
Memory, Attention and Decision Processes	8.54 (−14.45–31.52)	0.463
Social/Professional Role and Identity	0.96 (−45.62–47.55)	0.967

Furthermore, in the multivariate model, as shown in Table 4, both knowledge and intentions domains of the TDF were found to be significant predictors of physical inactivity among university PhD students, with the adjusted R^2^ = 0.51.

**Table 4:** Multiple regression analysis for predictors of Physical Inactivity among PhD students

Variables	β (95%CI of β)	P Value
Knowledge	12.39 (0.21–24.56)	0.046
Intentions	70.04 (37.26–102.82)	0.000
Physical Skills	−1.86 (− 25.98–22.56)	0.879
Goals	14.74 (−0.75–30.23)	0.062
Environmental Context and Resources	− 29.71 (−71.23–11.82)	0.159
Social Influences	−12.36 (−30.08-5.35)	0.169
Emotion and Reinforcement	3.31 (−24.56–31.18)	0.814
Beliefs about Capabilities	1.53 (− 25.74–28.80)	0.912
Beliefs about Consequences	84.23 (−10.78–179.25)	0.082
Behavioural Regulation	3.20 (− 5.63–12.03)	0.474
Cognitive and Interpersonal Skills	−17.69 (−64.44–29.07)	0.455
Optimism	24.06 (−2.40–50.51)	0.074
Memory, Attention and Decision Processes	−4.59 (−23.13–13.95)	0.624
Social/Professional Role and Identity	−10.58 (−44.66–22.96)	0.526

Lower knowledge score (β = 12.39; 95% CI = 0.21–24.56; *p* = 0.046) and lower intentions score (β = 70.04; 95% CI = 37.26–102.82; *p* = 0.001) were both associated with higher total self-reported physical inactivity among the PhD students. Overall, in the regression models, the independent variables explained 25 and 51% of the variance of the total self-reported physical inactivity in university administrative staff and PhD students, respectively (see Table 4).

#### Psychometric properties of the surveys

The Determinants for Physical Activity Questionnaire (DPAQ) was the only validated survey that was used to collect data in this present study. Since no validation studies were found to measure the other domains of the TDF, i.e., reinforcement social/professional role and identity; and memory, attention, and decision processes domains, which were measured using six additional items, their psychometric properties were measured using data from the present study.

The ‘reinforcement’ domain of the TDF was measured using a 4-item intrinsic motivation subscale of the Motivation for Physical Activity and Exercise/Working Out Questionnaire (MPAQ) [42]. Reliability tests among 121 administrative staff and 114 PhD students in the present study revealed excellent internal consistency, with Cronbach’s alpha coefficients of 0.94 and 0.95 for the administrative staff and PhD students, respectively, indicating that these items reliably assess the ‘reinforcement’ domain of the TDF. The items in this subscale were evaluated with a 7-point Likert Scale varying from 1= ‘Not at all true’ to 7 = ‘Very True’. The scores of items in this subscale were then summed up and divided by the number of items to get the mean score.

The ‘social/professional role and identity’ and ‘memory, attention & decision processes’ domains of the TDF were measured using six additional items (i.e., three items to measure each of the two domains) developed through an iterative process between the lead author and the research project team. The development of these additional six items is reported elsewhere [40]. Reliability tests on the data obtained from the 121 administrative staff and 114 PhD students who took part in a study to validate these six items revealed good internal consistency, with Cronbach’s alpha coefficients of 0.75 and 0.81 for the administrative staff and PhD students, respectively, suggesting that these six items consistently assess ‘memory, attention & decision processes’ and ‘professional/social role and identity’ domains of the TDF. These six items were evaluated with a 7-point Likert scale varying from 1 = ‘Strongly agree’ to 7 = ‘Strongly disagree’. For example, ‘Sometimes I just forget I had planned to do physical activity, because I am busy doing something else’ demonstrates the ‘memory, attention and decision processes’ domain; ‘Being physically active is seen to be an important attribute for someone in my job role/people in my course’ demonstrates the ‘professional/role and identity’ domain. The scores of the three items for each of the two domains were then separately summed up and divided by the total number of items to get the mean scores.

## Discussion

The aim of this study was to examine the predictors of physical inactivity among inactive university administrative staff and PhD students, using the TDF and to provide intervention targets for future interventions aimed at changing behaviour towards PA in the university setting.

In this present study, 64% of administrative staff and 62% of PhD students who responded to the survey were physically inactive (i.e., not meeting the recommended PA guidelines of 150 minutes of moderate to vigorous PA weekly) and thus the sample of focus. Interestingly, the student percentages are higher than that those reported by Pengpid et al. [18] who suggested a global physical inactivity prevalence of 41%, (varying from 22 to 81%) among students across universities in 23 low-, medium- and high-income countries.. As with university students, previous research revealed that 42% [19] to 59% [20] of university staff were physically inactive, which was lower than the levels reported by the administrative staff in this present study. The higher physical inactivity levels reported in this present study in comparison with other similar studies may be due to diverse population sizes, participant characteristics, and settings; different perceived levels of beliefs in the health benefits of PA; different levels of individual mastery of exercise; different cultural and environmental factors; as well as the diverse instruments used to assess PA in these studies [18, 21]. This is reinforced by Murphy et al. [43] indicating that these variations in physical inactivity levels reported in universities globally may be explained by the diverse instruments used to assess PA levels within studies. For example, while this current study used the GPAQ to measure PA levels among administrative staff and PhD students in a single university setting, Pengpid et al. [18] used the International Physical Activity Questionnaire (IPAQ) to measure PA among university undergraduate students in 24 universities across 23 countries. In support of this, a recent systematic review by Garcia-Alverez and Faubel [44], aimed at assessing the instruments used to measure PA in the university setting, revealed that diverse self-report questionnaires were used to measure PA, thus making comparisons across different studies challenging [44]. Therefore, more studies that use standardised self-report instruments such as the GPAQ to measure PA levels in the university setting, as well as other settings are needed to improve comparability across studies. Additionally, objective measures such as accelerometers and pedometers may be used to measure PA in the university setting to improve precision of measurements and comparability across studies [7].

The TDF was employed in this present study to identify the predictors of physical inactivity amongst the university administrative staff and PhD students that this study had identified as physically inactive. The study found that the ‘physical skills’ domain of the TDF (i.e. abilities or proficiencies gained through practice) was the only significant predictor of physical inactivity amongst university administrative staff, as also reported in a study by Flannery et al. [32] for a different population (overweight and obese pregnant women). The lack of physical skills to engage in PA may reduce peoples’ self-efficacy and thus motivation to engage in PA, thereby increasing their inactivity levels [45]. Therefore, based on the current study’s finding, it is recommended to improve individuals’ physical literacy as well as fundamental motor skills through supervised PA or exercise to enhance self-efficacy to participate in PA which may improve their PA engagement [46]. The current study is the first to assess predictors of physical inactivity in university staff using the TDF and it adds to existing research that used such TDF-based assessments among diverse populations and settings. The diversity of populations and settings may arguably limit the transfer of knowledge but this growing body of research in fact provides important insights that can inform future research and interventions. For example, a study by Haith-Cooper et al. [31], aimed at assessing barriers and enablers to PA and exercise among asylum seekers using the TDF, indicated that the lack of physical skills to engage in PA was a major reason for physical inactivity in this group. Similarly, Quigley et al. [33] using the TDF, demonstrated that lack of physical skills was a major reason for physical inactivity among older adults with HIV. Further large-scale studies in the university setting are needed to replicate the relationship between physical skills and physical inactivity levels among university staff revealed in the current study.

The findings of the current study also suggested that the ‘knowledge’ and ‘intentions’ domains of the TDF were significant predictors of physical inactivity amongst university PhD students. These findings support previous evidence [31, 47] that lack of knowledge is a major predictor of physical inactivity, possibly because most people are unaware of the harmful effects of physical inactivity and the recommended levels of PA required to gain health benefits, and may thus not perceive the necessity to participate in PA [47]. This is aligned with the findings of a cross-sectional study by Abula et al. [48] suggesting that knowledge about PA recommendations was associated with higher levels of PA among university students. Knowledge about PA recommendations is generally low among university students [48–51] which has been linked with an increase in physical inactivity levels. Therefore, in addressing the prevalence of physical inactivity in universities, future interventions should consider increasing students’ knowledge about PA by incorporating information about recommended PA levels and how to achieve them; specific health problems linked with physical inactivity; and benefits that can be gained through participation in routine PA in health promotion materials.

Changes in intention to engage in a behaviour does not always translate to changes in the behaviour (intention-behaviour gap) [52], with people with strong intentions about a given objective not regularly accomplishing them successfully [53, 54]. This is relevant to this current study and research on intentions, because understanding the intention-behaviour gap will help in the development of interventions that will improve the translation of intentions (e.g. willingness to participate in PA) into behaviour (e.g. actually participating in PA) [52]. A major reason for this intention-behaviour gap is probably due to the lack of effective cognitive or executive control (i.e. the ability of peoples to coordinate and focus their thoughts towards carrying out an intended behaviour) [55]. Likewise, setting optimistic goals, competing goals, unwanted thought, anxiety, and peoples’ socioeconomic status have also been established to contribute to the intention-behaviour gap [52].

Furthermore, Orbell & Sheeran [56] posit that this inability to accomplish a goal, in spite of strong intentions, may be because the stages of developing and implementing certain intentions are, in reality, two distinct processes. These stages are the motivational stage (where the positive intention to enact a behaviour is formed) and the volitional stage (where an individual enacts the previously formed intention) [57]. The concept of implementation intentions was thus developed grounded on the volitional stage [53]. Implementation intentions postulate that people are more likely to perform a behaviour if they form plans of where, when and how to perform the behaviour [57]. This is because implementation intentions creates a link between the mental illustrations of certain reminders (beneficial or unsafe situations) and means of accomplishing goals (mental or behavioural reactions) in an act of self-determination, thereby prompting people to complete their set goals [57]. Therefore, future interventions to promote PA in both universities and other settings should employ strategies such as the use of implementation intentions templates to plan when, where, and how individuals intend to participate in PA, as well as to plan how to overcome potential barriers, to enhance peoples’ intentions to engage in PA.

In this study, there were different predictors of physical inactivity amongst the administrative staff (lack of physical skills) and PhD students (lack of intentions and knowledge) as hypothesised. However, since no study, to our knowledge, has assessed the predictors of physical inactivity among university staff and/or students using the TDF, it was challenging to identify the reasons for the differences in predictors of physical inactivity between university administrative staff and PhD students. Therefore, it is important for future interventions to focus on identifying the reasons for the reported differences in predictors of physical inactivity between university administrative staff and PhD students.

There were some limitations which should be considered in interpreting the results of this study. Firstly, self-report questionnaires were used to assess physical inactivity levels, which may be susceptible to underestimation, overestimation, social desirability and recall biases [58–60]. However, self-report measures are widely used because they are economical, not difficult to administer and do not change behaviour, and thus well-suited for large-scale studies [58]. Moreover, objective measures such as pedometers and accelerometers are ideal but are both costly and difficult to administer in large-scale investigations [61]. Self-report measures are largely used by studies in this field, making it easy to compare results across different studies. Although the anonymity of the surveys reduces the problem of recall and social desirability bias, future studies should nevertheless contemplate using objective instruments such as pedometers or accelerometers to assess physical inactivity levels. Another limitation of the present study was the higher percentage of female administrative staff (84.3%) who were mainly white (94%), and higher percentage of male PhD students (55.3%) who were also mainly white (71.1%), which may have masked the findings; future studies will need to establish if the current studies generalise to a broader population. As a result of the format and anonymity of the online questionnaires, regulating the proportion of male and female respondents was challenging. Future studies should consider putting measures in place, such as carrying out telephone surveys, to ensure a more even representation of gender among participants. Finally, even though the number of the participants recruited did not meet the minimum as calculated by the power analysis, and may be seen as a limitation, the findings of this study still provide valuable information about the predictors of physical inactivity amongst university administrative staff and PhD students. As a result of this, the findings of this study may not be representative of these populations and should therefore be interpreted with caution. Despite these limitations, this is the first study carried out in a university setting to examine the predictors of physical inactivity among administrative staff and PhD students, using the theoretical domains framework. More studies are needed to confirm the findings of this present study.

## Conclusions

The current study provides an overview of the behavioural factors inhibiting PA in a university setting. Utilising the TDF was shown to be a sound theoretical starting point for understanding the predictors of physical inactivity in university settings. This study suggests that physical inactivity levels were high among university administrative staff and PhD students. These findings necessitate well planned interventions by suitable authorities in the institution of higher education. The current study also identified a strong association between lack of physical skills and physical inactivity among administrative staff, and strong associations between lack of knowledge and intentions, and physical inactivity among PhD students. Future investigations are recommended to identify and evaluate suitable health campaign approaches. Future research in universities, as well as other settings, that aim to promote pro-physical activity behaviours should endeavour to focus on physical skills, knowledge and intentions when designing health promotion strategies.

## Data Availability

The datasets (i.e., survey responses and SPSS outputs) generated and analysed during this current study are available in the Mendeley Data repository: 10.17632/j9gmgb9txx.1

## Abbreviations

DPAQ: Determinants of Physical activity questionnaire
GPAQ: Global physical activity questionnaire
MET: Metabolic equivalent
PA: Physical activity
PhD: Doctor of Philosophy
TDF: Theoretical domains framework
UK: United Kingdom.
